# Sepsis-induced myocardial dysfunction: pathophysiology and management

**DOI:** 10.1186/s40560-016-0148-1

**Published:** 2016-03-23

**Authors:** Yasuyuki Kakihana, Takashi Ito, Mayumi Nakahara, Keiji Yamaguchi, Tomotsugu Yasuda

**Affiliations:** Department of Emergency and Intensive Care Medicine, Kagoshima University Graduate School of Medical and Dental Sciences, 8-35-1 Sakuragaoka, Kagoshima, 890-8520 Japan; Department of Systems Biology in Thromboregulation, Kagoshima University Graduate School of Medical and Dental Sciences, Kagoshima, Japan; Department of Anesthesiology and Critical Care Medicine, Kagoshima University Graduate School of Medical and Dental Sciences, Kagoshima, Japan

**Keywords:** Damage-associated molecular patterns, Immune system, Infection, Septic shock, Systemic inflammatory response syndrome

## Abstract

Sepsis is aggravated by an inappropriate immune response to invading microorganisms, which occasionally leads to multiple organ failure. Several lines of evidence suggest that the ventricular myocardium is depressed during sepsis with features of diastolic dysfunction. Potential candidates responsible for septic cardiomyopathy include pathogen-associated molecular patterns (PAMPs), cytokines, and nitric oxide. Extracellular histones and high-mobility group box 1 that function as endogenous damage-associated molecular patterns (DAMPs) also contribute to the myocardial dysfunction associated with sepsis. If untreated, persistent shock causes cellular injury and the liberation of further DAMPs. Like PAMPs, DAMPs have the potential to activate inflammation, creating a vicious circle. Early infection control with adequate antibiotic care is important during septic shock to decrease PAMPs arising from invasive microorganisms. Early aggressive fluid resuscitation as well as the administration of vasopressors and inotropes is also important to reduce DAMPs generated by damaged cells although excessive volume loading, and prolonged administration of catecholamines might be harmful. This review delineates some features of septic myocardial dysfunction, assesses its most common underlying mechanisms, and briefly outlines current therapeutic strategies and potential future approaches.

## Introduction

Sepsis has been defined by consensus as a systemic inflammatory response syndrome (SIRS) to infection [1, 2]. It is generally viewed as being aggravated by an inappropriate immune response, and it occasionally leads to multiple organ failure and shock. The pathophysiology of septic shock is thought to involve complex interactions between pathogens and a host immune system. Recent advances in the molecular biology of sepsis have shown that the host immune system recognizes infection through recognition of pathogen-associated molecular patterns (PAMPs), such as lipopolysaccharide (LPS), lipoteichoic acid, flagellin and DNA in bacteria, mannan in fungi, and single- or double-stranded RNA in viruses. These mediators bind to pattern-recognition receptors (PRRs), such as toll-like receptors (TLRs) that are expressed on the surface of host cells. These PRRs are essential for initiating host immune defenses against invading pathogens and mediating PAMP recognition. They also serve as receptors for endogenous danger signals by identifying various damage-associated molecular patterns (DAMPs) as potent activators of the innate immune system [3–5]. The proinflammatory response induced by infection is normally balanced by anti-inflammatory cytokines. However, the normally effective inflammatory response to infection becomes systemically dysregulated during sepsis due to significantly imbalanced cytokine responses referred to as a cytokine storm. Ten TLRs have been identified in the human genome [6], and interactions between TLRs and PAMPs activate intracellular signal-transduction pathways that lead to the nuclear translocation of nuclear factor-κB (NF-κB) and the increased transcription of inflammatory mediators [7]. Among these, proinflammatory cytokines such as tumor necrosis factor-alpha (TNF-α) and interleukin-1-beta (IL-1β), chemokines, and lipid mediators play major roles in the inflammatory process [8]. The production of excess antimicrobial products and inflammatory mediators elicits the generation of reactive oxygen and nitrogen species, superoxide anion (O_2_^−^), and nitric oxide (NO), causing adjacent tissue damage and an amplified inflammatory reaction [9, 10]. The DAMPs released during tissue damage include heat-shock proteins, high-mobility group box 1 (HMGB1), histones, and oxidized lipoproteins. Other cytosolic constituents such as adenosine triphosphate (ATP) and mitochondrial products, including mitochondrial DNA (mtDNA), can also contribute to the activation of innate immunity that initiates SIRS and a sepsis-like state. Excessive production of DAMPs can activate inflammation, create a vicious circle, and finally facilitate cardiac dysfunction, multiple organ failure (MOF), and death. This review describes some important features of septic myocardial dysfunction, assesses the key underlying mechanisms of cardiac dysfunction in sepsis, and briefly outlines current therapeutic strategies and potential future approaches.

## Review

### Pathophysiology of septic shock and secondary myocardial dysfunction

**Fig. 1:**
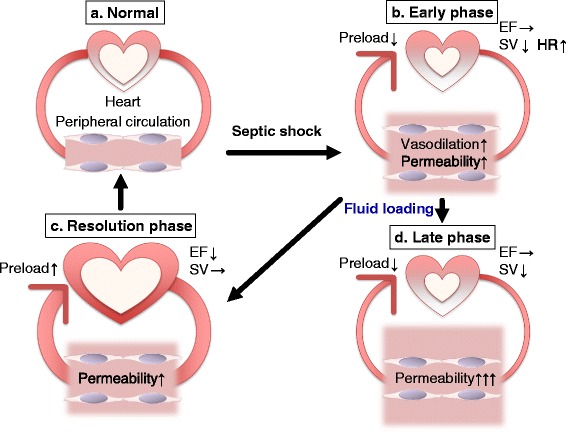
Pathophysiology of septic shock and secondary myocardial dysfunction. (*a*) In the normal condition, cardiac output is adequate to meet the oxygen demand in peripheral tissues. (*b*) At the very early phase of sepsis, LV ejection fraction (EF) is not impaired (typically LVEF >55 %), but stroke volume (SV) is low because of insufficient cardiac preload due to a high vascular permeability and vasodilation. The compensatory increase in heart rate (HR) is often insufficient to maintain adequate cardiac output. (*c*) After fluid loading, SV can be recovered especially in the case of survivors while LVEF is temporarily decreased (typically <45 %) in part due to high LVEDV. This indicates that low LVEF may represent preload optimization and good adaptation. (*d*) During the later phase of sepsis, non-survivors are given more fluid than survivors but, nevertheless, have lower LVEDV suggesting a persistent vascular hyperpermeability and preload deficiency. In these cases, LVEF can be retained in part due to low LVEDV and/or ongoing harmful adrenergic over-stimulation

Septic distributive shock is a circulatory maldistribution associated with peripheral vasodilation, as well as arterial and capillary shunting. However, the pathophysiology of septic shock comprises both warm (hyperdynamic) and cold (hypodynamic) types. The early phase of septic shock is called hyperdynamic, or warm shock, that is characterized by high cardiac output, low peripheral vascular resistance, and warm extremities (Fig. 1(a–c)). The late phase comprises concomitant hypotension followed by hypodynamic, or cold shock, with low cardiac output, poor peripheral perfusion, cool extremities (Fig. 1(d)), and finally, death [11–13]. Inadequate resuscitation, relative hypovolemia, and an increased afterload were initially thought to be the hemodynamic profile of patients with hypodynamic shock [14, 15]. Adequate volume resuscitation and the profoundly reduced systemic vascular resistance typically encountered in patients with sepsis lead to a normal or elevated cardiac index [16]. However, despite increased cardiac output and a normal stroke volume, myocardial dysfunction is significant in patients with septic shock. Notably, ejection fraction (EF) is lower and end-diastolic volume (EDV) is higher in survivors, compared to non-survivors of shock. This suggests that ventricular dilation might be a compensatory mechanism to maintain adequate cardiac output and protect against myocardial depression [17]. A recent study of 90 patients with septic shock identified global left ventricular (LV) hypokinesia in 51 % of patients during the first 48 h of treatment [18]. They also found that patients who died had a significantly stop higher left ventricular ejection fraction (LVEF) and a significantly lower left ventricular end-diastolic volume (LVEDV) than those who recovered; the latter were insensitive to volume loading (Fig. 1(c, d)). Other studies of septic shock lasting 48 h have found that 24 to 44 % of patients had systolic LV dysfunction and a further 44 % had echocardiographic features of diastolic dysfunction [19–21]. These EF abnormalities are reversible, with full recovery of cardiac function at 7 to 10 days after the onset of sepsis. However, more fluids were administered during the first 24 h of intensive care, and the overall mortality rate was higher among patients with myocardial depression than in those without myocardial dysfunction [21]. Importantly, cardiovascular dysfunction in sepsis is associated with a significantly increased mortality rate of 70–90 % compared with 20 % among patients with sepsis that is not accompanied by cardiovascular impairment [22]. Myocardial edema due to inflammation-induced vascular leakage might also influence cardiac compliance and function [23, 24]. In addition, ventricular function is influenced by changes in afterload. Pulmonary hypertension will worsen right-heart function [25], whereas right-heart dilation will impair left-heart function [26]. Endothelial cells producing vasoactive molecules that regulate peripheral vascular resistance are impaired during septic shock, and thus, endothelial dysfunction plays a crucial role in its pathophysiology [27]. This is because impaired endothelium-derived NO release could alter the physiological regulation of blood flow distribution via coronary vasospasm combined with an increase in peripheral vascular resistance and the associated elevation of cardiac workload and myocardial oxygen demand.

In conclusion, despite high LVEF (typically >55 %), stroke volume at the very early phase of sepsis is low because of insufficient cardiac preload due to a high vascular permeability and vasodilation (Fig. 1(b)). The compensatory tachycardia is often insufficient to maintain adequate cardiac output during this very early phase of sepsis, as demonstrated by elevated lactate levels. After fluid loading, LVEF was markedly decreased (typically <45 %) in all patients during the first 3 days of hemodynamic support (Fig. 1(c)). However, LV systolic dysfunction is common in septic patients and potentially reversible in survivors. During the later phase of sepsis, non-survivors were given more fluid than survivors but, nevertheless, had lower LVEDV suggesting a persistent preload deficiency (Fig. 1(d)). Some studies reported more cardiac depression in sepsis survivors compared to non-survivors [17, 18]. How can such conflicting results be explained? In very severe septic patients, the presence of profound myocardial depression defined by a low LVEF may represent preload optimization and good adaptation, while a normal LVEF could be caused by persistent preload deficiency and/or ongoing harmful adrenergic over-stimulation (Fig. 1(c, d)).

### Global ischemia and myocardial dysfunction in sepsis

Early sepsis and septic shock are characterized by circulatory abnormalities that are usually related to intravascular volume depletion and vasodilation. This potentially causes an oxygen supply–demand imbalance in various organ beds [28], and cardiac performance is likely to be reduced in insufficiently resuscitated animal models [29–31]. Therefore, earlier theories suggested that global myocardial ischemia might be responsible for myocardial dysfunction in sepsis. However, Cunnion et al. found in a study of coronary sinus catheterization that coronary flow was the same or greater in patients with septic shock compared with normal individuals. Although all of these findings reflect important changes in coronary flow and myocardial metabolism, and mirror the effects in the peripheral circulation during sepsis, evidence does not support the notion that global ischemia is an underlying cause of myocardial dysfunction in sepsis. Macrocirculatory coronary blood flow is increased in patients with established septic shock [32, 33], but cardiac microcirculation undergoes major changes during sepsis with endothelial disruption and blood flow maldistribution [34]. Heterogeneous cardiac microvascular blood flow, swollen endothelial cells, and non-occlusive intravascular fibrin depositions have been found in the hearts of dogs with endotoxemia [35, 36]. In addition, circulating neutrophils migrate into the interstitium [37]. These findings indicated that changes in the distribution of flow were localized to areas of ischemia and that this could explain the occasional appearance of elevated troponin levels associated with the severity of cardiac dysfunction [38]. However, Hotchkiss et al. [39] did not find cellular hypoxia in the hearts of rats with sepsis using the marker [18F]fluoromisonidazole. The current belief is that increases in plasma troponin are due to increased membrane permeability induced by myocardial cytokines, although this remains a matter of debate. As in the peripheral circulation, these changes could be attributed to disrupted flow autoregulation or oxygen utilization [40, 41]. Several magnetic resonance studies have identified normal levels of high-energy phosphate in the myocardium of animal models of sepsis [42, 43]. In addition, myocardial dysfunction in sepsis might reflect a hibernating myocardium [44]. The adequate O_2_ supply in sepsis suggests that myocardial depression is not related to tissue hypoperfusion but rather to circulating depressant factors or other mechanisms. Endothelial damage and induction of the coagulatory system also contribute to the pathophysiology of septic cardiomyopathy.

### Direct myocardial depression in sepsis

**Fig. 2:**
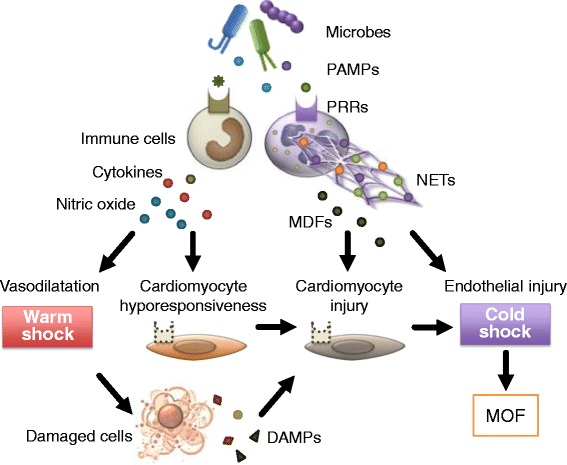
Direct myocardial depression in sepsis. A major mechanism of direct cardiac depression in sepsis is cardiomyocyte hyporesponsiveness due to down-regulation of β-adrenergic receptors and depression of post-receptor signaling pathways. These changes seem to be mediated by many substances, including cytokines and nitric oxide. Another mechanism of direct cardiac depression is cardiomyocyte injury or death, which can be induced by toxins, complements, damage-associated molecular patterns (DAMPs), neutrophil extracellular traps (NETs), and as-yet-unidentified myocardial depressant factors (MDFs). *MOF* multiple organ failure, *PAMPs* pathogen-associated molecular patterns, *PRRs* pattern recognition receptors

A major mechanism of direct cardiac depression in sepsis is the attenuation of the adrenergic response at the cardiomyocyte level due to down-regulation of β-adrenergic receptors and depression of post-receptor signaling pathways. These changes seem to be mediated by many substances, such as cytokines and nitric oxide. Another mechanism of direct cardiac depression in sepsis is cardiomyocyte injury or death, which can be induced by toxins, complements, DAMPs, and as-yet-unidentified myocardial depressants (Fig. 2).

#### Myocardial depressants

Numerous bacterial toxins as well as primary, secondary, and final mediators are usually involved in the pathogenesis of systemic inflammation. A myocardial depressant factor (MDF) was discovered in an experimental animal model of hemorrhagic shock during 1947 [45]. The MDF determined in the blood of dogs during induced endotoxic shock seemed to be an 800–1000 dalton peptide that originated in the pancreas [46]. Parrillo et al. [47] quantitatively linked the clinical degree of septic myocardial dysfunction with the effect of serum from septic patients on rat cardiac myocytes during 1985; clinical severity correlated with a decrease in the extent and velocity of myocyte shortening. These effects were notably absent when serum was applied that had been sourced from patients who were convalescing from sepsis or who were critically ill but without sepsis. Several MDFs have been identified, although the chemical composition of others remains unknown [48–50]. Nevertheless, the combination of TNF-α and IL-1β is extremely cardiodepressive [51]. Administering recombinant TNF-α to animal models elicits fever, lactic acidosis, hemodynamic changes, and even death. Many studies of anti-TNF-α antibodies in humans and other animals have found a rapid improvement in cardiovascular parameters but no decrease in mortality [52, 53]. Cytokines (TNF-α and IL-1β) might play key roles in the early decrease in contractility, but they cannot explain the prolonged myocardial dysfunction in sepsis because the effect of TNF-α is maximal between 8 and 48 h after administration [54]. Both TNF-α and IL-1β induce the release of additional factors (such as NO) that in turn alter myocardial function [55, 56]. A constellation of factors rather than any individual factor might influence the onset of sepsis-induced myocardial dysfunction through the release, activation, or inhibition of other cellular mediators.

#### Cytokines and nitric oxide

Both TNF-α and IL-1β are primary players in the hierarchy of proinflammatory mediator cascades [57], whereas nitric oxide (NO) [58] and oxygen-free radicals [59] are secondary effectors in the setting of SIRS cardiodepression. Sepsis leads to the expression of inducible NO synthase (iNOS) in the myocardium [60, 61] followed by high levels of NO production. This consequently contributes to myocardial dysfunction and increases total levels of sarcoplasmic reticulum Ca^2+^ and myofilament sensitivity to Ca^2+^ [62], partly through the generation of cytotoxic peroxynitrite from a diffusion-controlled reaction between NO and another free radical, superoxide. Sepsis-induced myocardial depression can be prevented in vitro by administering nonspecific NOS inhibitors, for example, inhibitors of guanylate cyclase such as *N*-methyl-l-arginine and methylene blue [63]. Infusing methylene blue into patients with sepsis strikingly improves mean arterial pressure, stroke volume, and left ventricular stroke work and decreases the requirement for inotropic support. Yet, the outcomes remain unaltered [64]. Conflicting results from studies of selective and nonselective iNOS inhibition indicate that constitutive NOS isoforms, such as neuronal (nNOS) and endothelial (eNOS), have potential roles in regulating cardiomyocyte homeostasis and function. These constitutive NOS isoforms may play an important role in the very early phase of myocardial depression. Myocardial eNOS in the sarcolemmal membrane produces NO that modifies L-calcium channels to inhibit calcium entry and induces myofibril relaxation, which might play an important protective role against sepsis-induced myocardial dysfunction [65, 66]. Neuronal NOS is a component of the central and peripheral nervous systems, and it is constitutively expressed in cardiac myocytes. Several studies have shown that nNOS can regulate the β-adrenergic receptor pathway [67]. A functional NOS that was recently identified in red blood cells (rbcNOS) regulates the deformability of erythrocyte membranes and inhibits platelet activation in sepsis [68]. Since many NOS isoforms have various modulating interactions and dose-dependent NO effects and given the precise balance among NO, superoxide, and thus peroxynitrite generated in subcellular compartments, further advances in understanding the complexity of NO biology and its derived reactive nitrogen species offer the promise of new, more specific, and effective therapeutic targets.

#### Mitochondrial dysfunction

Since the heart is rich in mitochondria that are not only involved in energy provision but also in intracellular calcium regulation, the degree of mitochondrial dysfunction is tightly linked to sepsis-induced cardiac dysfunction and prognosis [69–71]. The activities of complexes I and II of the mitochondrial respiratory chain are diminished in hearts from animals with sepsis [72, 73], and this might be due to the detrimental effects of sepsis mediators such as NO [74], TNF-α, IL-1β [75], and others. Mitochondrial permeability transition pores might also play a role in the development of mitochondrial dysfunction [76]. Reactive oxygen species (ROS) such as superoxide and NO suppress mitochondrial function during sepsis. This ultimately causes an increase in mitochondrial mass due to internal edema within mitochondria that is often associated with their dysfunction. One theory suggests that sepsis-induced myocardial dysfunction could represent a protective adaptation to reduced energy consumption during a state of low levels of ATP produced by dysfunctional mitochondria. This is similar to the phenomenon of the hibernating myocardium during ischemia. Recent studies have found that mitochondria generate a significant amount of DAMPs [77], including mtROS, mtDNA fragments, ATP [78, 79], and cytochrome C [80, 81]. These molecules are released from fragmented mitochondria into the circulatory system during cell death and organ damage, initiating inflammatory responses through multifactorial pathways.

#### DAMPs: histones and HMGB1

Extracellular histones function as endogenous DAMPs that might interact with TLR2 and TLR4 on various cell types, including cardiomyocytes to reduce mitochondrial membrane potential and ATP levels. These activities cause cell damage, the dysfunction of organs including the heart, and lethality [82–84]. Extracellular histones appear to arise in a complement (C5a)-dependent fashion related to neutrophil activation that results in neutrophil extracellular traps (NETs) [85]. Exposing cardiomyocytes to histones in vitro results in obvious [Ca^2+^]i elevation in cardiomyocytes and a loss of homeostasis in the redox system and in [Ca^2+^]i, as well as defects in mitochondrial function due to increased membrane permeability [86]. We did not detect histone H3 in plasma from healthy volunteers but found significant levels in patients with sepsis and disseminated intravascular coagulation (DIC), especially in those who did not survive [87]. Alhamdi et al. [88] showed similar findings, and they also discovered that circulating histone concentrations closely correlate with elevated levels of cardiac troponin T (cTnT) in patients with sepsis, which probably contributes to septic cardiac events and mortality. They concluded that circulating histones are novel and important mediators of septic cardiomyopathy that could play prognostic and therapeutic roles.

The proinflammatory mediator HMGB1 also mediates endotoxin lethality and plays an important role in the pathogenesis of cardiac dysfunction and many other diseases. Zhang et al. [89] showed that at least one mechanism underlying HMGB1-induced cardiac dysfunction is the increased level of intracellular ROS induced through HMGB1–TLR4 interaction and consequently enhanced oxidative stress and Ca^2+^/calmodulin-dependent protein kinase (CaMKII)-activated phosphorylation in ryanodine receptor 2 (RyR2). Furthermore, HMGB1 enhances a Ca^2+^ spark-mediated sarcoplasmic reticulum (SR) Ca^2+^ leak through the TLR4–ROS signaling pathway, which partially depletes the SR Ca^2+^ content and impairs cardiac excitation–contraction (EC) coupling. Hence, systolic Ca^2+^ transients and myocyte contractility are decreased. Inhibiting TLR4 or adding an antioxidant prevents enhancement of the SR Ca^2+^ leak, resulting in improved cardiac EC coupling. Preventing the SR Ca^2+^ leak might serve as a potential therapeutic strategy with which to treat cardiac dysfunction associated with HMGB1 overproduction. In conclusion, circulatory DAMPs (histone and/or HMGB1) directly injure myocytes or damaged myocytes release these DAMPs, resulting in myocardial dysfunction.

### Management of myocardial dysfunction in septic shock

**Fig. 3:**
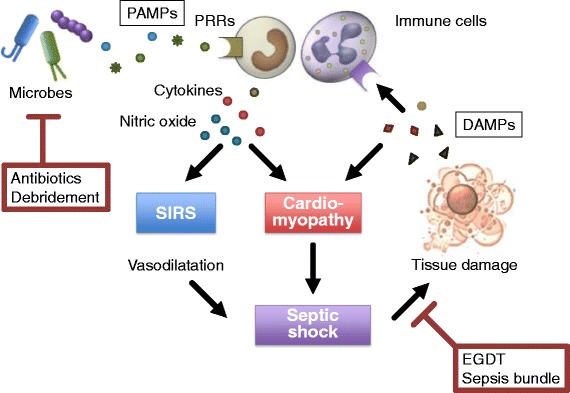
Management of myocardial dysfunction in septic shock. Prompt and adequate antibiotic therapy, accompanied by surgical removal of the infectious focus if indicated and feasible, is important to decrease PAMPs arising from invasive microorganisms. Early goal-directed therapy (EGDT), including fluid resuscitation, vasopressor and inotropic therapy, and red blood cell transfusion, is important to re-establish organ perfusion pressure, which helps maintain blood flow to tissues and reduces the release of damage-associated molecular patterns (DAMPs) in patients with septic shock. Sepsis bundle is a selected set of elements of care distilled from Surviving Sepsis Campaign guidelines. *PAMPs* pathogen-associated molecular patterns, *PRRs* pattern recognition receptors, *SIRS* systemic inflammatory response syndrome

Prompt and adequate antibiotic therapy, accompanied by surgical removal of the infectious focus, if indicated and feasible, is the mainstay and only strictly causal line of therapy for sepsis. The optimal treatment for myocardial dysfunction includes the proper management of infection and the optimization of hemodynamic parameters. Early control of the source and monitoring hemocultures in conjunction with early adequate antibiotic care is important to decrease PAMPs arising from invasive microorganisms (Fig. 3). Moreover, aggressive fluid replacement guided by monitoring fluid response parameters appears to be a rational strategy to remedy hypovolemia. While early and sufficient fluid administration is likely to be beneficial, excessive volume loading is harmful. The risk of pulmonary edema formation is particularly elevated due to increased permeability of the pulmonary microcirculation and LV diastolic dysfunction. Supportive therapy encompasses early and goal-directed fluid resuscitation, vasopressor and inotropic therapy, red blood cell transfusion, mechanical ventilation, and renal support when indicated. Goal-directed therapy (GDT) appears to significantly reduce overall mortality in patients with sepsis, especially when implemented within the first 6 h of admission; this is called early GDT (EGDT) [90]. Early supportive treatment is mandatory for severe sepsis and septic shock in addition to causal therapy; this is called Surviving Sepsis Campaign bundles [91]. Therefore, stabilizing arterial pressure as soon as possible is very important to re-establish organ perfusion pressure, which helps maintain blood flow to tissues and reduces the release of DAMP in patients with septic shock (Fig. 3). Norepinephrine is the vasopressor of choice when a patient is unresponsive to fluids. However, these efforts do not normalize hemodynamics in 10–20 % of patients with septic shock, indicating a high probability that sepsis-induced myocardial dysfunction diminishes cardiac output [92]. Patients with myocardial depression will require inotropic drugs to obtain adequate tissue perfusion and improve hemodynamics, and dobutamine is the first choice recommended by the Surviving Sepsis Campaign guidelines (SSCG) 2012 [93]. After optimization of volume status, cardiac output can be increased by inotropes. While early administration of catecholamines might be necessary to reverse shock and restore adequate organ perfusion, prolonged administration, particularly at unnecessarily high doses, might be harmful and exacerbate myocardial damage. Furthermore, myocardial depression causes a poor response to β-adrenergics in patients with septic shock. Myocardial β-adrenergic receptor density is decreased in rats with sepsis [94, 95], and stimulatory G-proteins are decreased in rabbits with endotoxemia [96], whereas inhibitory G-proteins are increased in both non-survivors of septic shock and in experimental animals with sepsis [97, 98]. These changes, namely down-regulation of the β-adrenergic response, result in decreased adenylate cyclase activity and reduced levels of cyclic adenosine monophosphate. Barraud et al. [99] showed that the calcium-sensitizing drug levosimendan at least partially restored cardiac contraction, relaxation, and filling without altering vascular properties in a model of human sepsis with myocardial dysfunction, whereas the cyclic adenosine monophosphate (cAMP)-dependent inotropes milrinone (a phosphodiesterase 3 inhibitor) and dobutamine did not. By contrast, both milrinone and dobutamine corrected systolic impairment but did not restore diastolic function. These findings confirmed that levosimendan works as a strategic therapy targeting cardiac abnormalities in patients with sepsis. However, no definitive studies have supported levosimendan as the optimal choice of medication for patients presenting with myocardial dysfunction due to sepsis, and its application to treat such patients has not been authorized in a few countries (including Japan).

Beta-blockers can prevent ischemia, decrease oxygen demand (by reducing cardiac output up to 20 % without worsening oxygen utilization or increasing lactate levels), and decrease TNF-α production [100], allowing for better preservation of cardiac function. Beta-blocking agents could be beneficial because evidence suggests that beta adrenergic stress is a major factor in the pathogenesis of sepsis-induced myocardial dysfunction [101]. The ultrashort-acting beta-blocker landiolol is associated with a significant reduction in serum levels of the inflammatory mediator HMGB1 and histological lung damage [102]. Gore and Wolfe [103] showed that esmolol, another ultrashort-acting beta-blocker, could reduce the risk of myocardial ischemia without the systemic consequences of hypoperfusion in patients with sepsis. Schmittinger and coworkers [104] found that combining milrinone with the enteral beta-blocker metoprolol maintained the cardiac index with a lower heart rate and a higher stroke volume index. Information about this issue in humans is scarce and controversy surrounds the notion that to administer a negative inotropic drug to a patient with sepsis-induced myocardial dysfunction is potentially deleterious. Recombinant thrombomodulin (rTM) has been approved for treating DIC in Japan, and it is currently undergoing a phase III clinical trial in the USA. In addition to its anti-coagulant role, rTM play a role in regulating DAMPs-mediated inflammation, in part through the neutralization of extracellular histones and HMGB1 [87, 105]. However, further detailed study is required to evaluate the effectiveness of rTM against histones or HMGB1-induced myocardial dysfunction in septic shock.

## Conclusions

The pathophysiology of sepsis-induced myocardial dysfunction has not yet been defined, and topics range from patho-mechanisms to treatment. In reality, only support treatment is available for patients with sepsis and no specific drug can reverse the associated sepsis-induced myocardial dysfunction. Therefore, prompt appropriate antibiotic therapy accompanied by surgical removal of the infectious focus is very important for decreasing PAMPs, and supportive treatment comprising early aggressive fluid resuscitation with concurrent vasopressors and inotropic therapy is mandatory for septic shock. The SSCG recommend these bundle therapies, through which the initial hyper-activation of the innate immune system characterized by sepsis might be controlled. New approaches to the treatment of sepsis and a deeper understanding of its mechanisms should help improve the prognosis of patients with myocardial dysfunction in the near future.

## Abbreviations

ATP: adenosine triphosphate
CaMKII: Ca^2+^/calmodulin-dependent protein kinase
cAMP: cyclic adenosine monophosphate
cTnT: cardiac troponin T
DAMPS: damage-associated molecular patterns
DIC: disseminated intravascular coagulation
EC: excitation–contraction
EDV: end-diastolic volume
EF: ejection fraction
EGDT: early GDT
eNOS: endothelial nitric oxide synthase
GDT: goal-directed therapy
HMGB1: high-mobility group box 1
IL-1β: interleukin-1-beta
iNOS: inducible NO synthase
LPS: lipopolysaccharide
LV: left ventricular
MDF: myocardial depressant factor
MOF: multiple organ failure
NF-κB: nuclear factor-κB
nNOS: neuronal nitric oxide synthase
NO: nitric oxide
O_2_^−^: superoxide anion
PAMPS: pathogen-associated molecular patterns
PRRs: pattern-recognition receptors
rbcNOS: red blood cells nitric oxide synthase
ROS: reactive oxygen species
rTM: recombinant thrombomodulin
RyR2: ryanodine receptor 2
SIRS: systemic inflammatory response syndrome
SR: sarcoplasmic reticulum
SSCG: Surviving Sepsis Campaign guidelines
TLRs: toll-like receptors
TNF-α: tumor necrosis factor-alpha
